# Cholesterol metabolites exported from human brain

**DOI:** 10.1016/j.steroids.2015.01.026

**Published:** 2015-07

**Authors:** Luigi Iuliano, Peter J. Crick, Chiara Zerbinati, Luigi Tritapepe, Jonas Abdel-Khalik, Marc Poirot, Yuqin Wang, William J. Griffiths

**Affiliations:** a Department of Medico-Surgical Sciences and Biotechnology, Sapienza University of Rome, corso della Repubblica 79, Latina 04100, Italy; b College of Medicine, Grove Building, Swansea University, Singleton Park, Swansea SA2 8PP, UK; c Department of Anesthesiology and Intensive Care, Sapienza University of Rome, vial del Policlinico 163, Rome 00161, Italy; d UMR 1037 INSERM-University Toulouse III, Cancer Research Center of Toulouse, and Institut Claudius Regaud, 31052 Toulouse, France

**Keywords:** MS, mass spectrometry, GC, gas chromatography, LC, liquid chromatography, BBB, blood brain barrier, CYP, cytochrome P450, 24S-HC, 24S-hydroxycholesterol, 26-HC, (25R)26-hydroxycholesterol, 7αH,3O-CA, 7α-hydroxy-3-oxocholest-4-enoic acid, 3β,5α-diHC-6O, 3β,5α-dihydroxycholestan-6-one, C-triol, cholestane-3β,5α,6β-triol, 4α-HC, 4α-hydroxycholesterol, 4β-HC, 4β-hydroxycholesterol, GP, Girard P, 7β-HC, 7β-hydroxycholesterol, 7O-C, 7-oxocholesterol, 25-D_3_, 25-hydroxyvitamin D_3_, 7α,25-diHCO, 7α,25-dihydroxycholest-4-en-3-one, 7α,26-diHCO, 7α,(25R)26-hydroxycholest-4-en-3-one, ROS, reactive oxygen species, 7α,26-diHC, 7α,(25R)26-dihydroxycholesterol, 7α,25-diHC, 7α,25-dihydroxycholesterol, HSD3B7, hydroxysteroid dehydrogenase 3B7, 3β-HCA, 3β-hydroxycholest-5-en-(25R)26-oic acid, 3β,7α-diHCA, 3β,7α-dihydroxycholest-5-enoic acid, CHD, coronary heart disease, Oxysterol, LC–MS, GC–MS, 24S-hydroxycholesterol

## Abstract

The human brain contains approximately 25% of the body’s cholesterol. The brain is separated from the circulation by the blood brain barrier. While cholesterol will not passes this barrier, oxygenated forms of cholesterol can cross the barrier. Here by measuring the difference in the oxysterol content of blood plasma in the jugular vein and in a forearm vein by mass spectrometry (MS) we were able to determine the flux of more than 20 cholesterol metabolites between brain and the circulation. We confirm that 24S-hydroxycholesterol is exported from brain at a rate of about 2–3 mg/24 h. Gas chromatography (GC)–MS data shows that the cholesterol metabolites 5α-hydroxy-6-oxocholesterol (3β,5α-dihydroxycholestan-6-one), 7β-hydroxycholesterol and 7-oxocholesterol, generally considered to be formed through reactive oxygen species, are similarly exported from brain at rates of about 0.1, 2 and 2 mg/24 h, respectively. Although not to statistical significance both GC–MS and liquid chromatography (LC)–MS methods indicate that (25R)26-hydroxycholesterol is imported to brain, while LC–MS indicates that 7α-hydroxy-3-oxocholest-4-enoic acid is exported from brain.

## Highlights

• Flux of more than 20 sterols into and out from human brain measured.
• 24S-hydroxycholesterol confirmed to be exported from brain at about 2–3 mg/24 h.
• Other sterols exported from brain include 5α-hydroxy-6-oxo-, 7β-hydroxy- and 7-oxo-cholesterol.

## Graphical abstract

**Figure:**
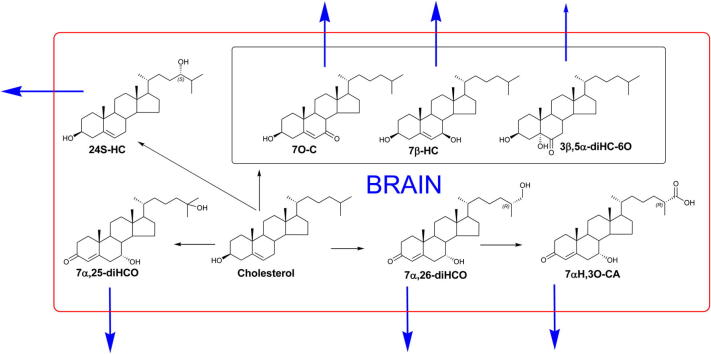

## 1 Introduction

The human brain contains about 25% of the body’s cholesterol, and cholesterol makes up about 2% of brain [1]. The brain is isolated from the circulation by the blood brain barrier (BBB) which is impermeable to cholesterol. The consequence of this is that essentially all brain cholesterol is synthesised from acetyl CoA in brain itself. The half life of cholesterol in human brain is about 5 years [2]. It is metabolised in brain by the cytochrome P450 (CYP) 46A1 enzyme to 24S-hydroxycholesterol (24S-HC, cholest-5-en-3β,24S-diol), which by virtue of the added hydroxy group to the cholesterol side-chain can pass the BBB and be exported from brain at a reported rate of about 4–7 mg/24 h [3–5]. It is believed that export of 24S-HC correspond to about 2/3 of cholesterol turn-over in brain of rodents, the origin of the remaining 1/3 has yet to be established [1]. In contrast to 24S-HC, (25R)26-hydroxycholesterol (26-HC) is reported to be imported to human brain at a rate of about 4–5 mg/24 h [5,6]. Note, we use the systematic nomenclature where addition of a hydroxy group to a terminal carbon atom of the cholesterol side-chain introducing R stereochemistry at C-25 results in the oxysterol named (25R)26-hydroxycholesterol [7]. The commonly used, but systematically incorrect, name for this compound is 27-hydroxycholesterol. Despite the high rate of import of 26-HC into brain the level of 26-HC in human brain (1–2 ng/mg) is much lower than that of 24S-HC (20 ng/mg) [8]. Interestingly, Meaney et al. have reported that the 26-HC metabolite 7α-hydroxy-3-oxocholest-4-enoic acid (7αH,3O-CA) is exported from brain at a rate of about 2 mg/24 h, accounting for much of the consumption of 26-HC in brain [9].

Gas chromatography (GC)–mass spectrometry (MS) and liquid chromatography (LC)–MS have been exploited widely for measurement of plasma or serum oxysterols [10–15]. In this work we have utilised both these methods measuring the levels of cholesterol metabolites in the jugular vein and a vein within the arm allowing us to measure the rate of flux of cholesterol metabolites out from, and into, brain.

## 2 Methods

### 2.1 Patient samples

Plasma was from Policlinico Umberto I, Rome, provided with written informed consent, institutional review board and ethical approval, and collected according to the principles of the Declaration of Helsinki. Sixteen males and two females were enrolled in the study. Sixty percent were current smokers, and 45% were also affected by type 2 diabetes and under oral antidiabetic medications. Patients were also taking medications which included antiplatelets, betablockers, angiotensin converting enzyme inhibitors, and statins. Export of cerebral oxysterols from brain was studied by measuring the gradient of concentration between the jugular vein (blood exiting the brain) and a forearm vein sample taken from 18 coronary heart disease patients undergoing on-pump myocardial revascularization surgery. Blood from the jugular vein was drawn in the intensive care unit through a 4F catheter, which was placed in the right jugular bulb via echoscan guide and position confirmed by neck X-ray.

Blood was collected in EDTA tubes; plasma was separated within 2 h of collection and stored at −80 °C until assay.

### 2.2 GC–MS analysis

Oxysterols were determined by GC–MS using deuterium-labelled internal standards as described by Dzeletovic et al. and Iuliano et al. [14,15]. In brief, 10 μL BHT in ethanol (5 mg/mL) and 50 μL EDTA (10 mg/mL) were added to a solution of 1 mL of plasma and 10 μL of ethanol containing deuterium labelled internal standards. Samples were subjected to alkaline hydrolysis (600 μL ethanolic KOH 5.9 M) for 2 h at room temperature with stirring. At the end of incubation, the solution was neutralised with 200 μL phosphoric acid and sterols extracted in chloroform:methanol (2:1, v/v). Solvent was evaporated under a stream of nitrogen, the residue dissolved in 1 mL of toluene, and oxysterols separated from cholesterol by solid phase extraction. Silica cartridges (100 mg), previously equilibrated with n-hexane, were loaded with toluene-dissolved samples. Cholesterol and non-cholesterol neutral sterols were eluted with 1% propan-2-ol in hexane before eluting oxysterol with 10% propan-2-ol in n-hexane. After removal of solvent, samples were converted to trimethylsilyl ethers by treatment with 130 μL Sylon HTP (hexamethyldisilylazane:trimethylchlorosilane:pyridine, 3:1:9) (Supelco, Bellafonte, PA) at 60 °C for 30 min. After incubation, the solution was evaporated under a stream of nitrogen, and the residue dissolved in n-hexane and transferred to an autosampler vial. Analyses were performed on an Agilent 6890N GC equipped with a 7683 series automatic liquid sampler, and interfaced with an Agilent 5973 Mass Spectrometer (Agilent Technologies; Palo Alto, CA). Separation was carried out on a 30 m capillary column (HP-5MS 30 × 0.25 mm ID, 0.25 μm thickness). Quantification of oxysterols was made by the isotope dilution method.

3β,5α-Dihydroxycholestan-6-one (3β,5α-diHC-6O), 3β,5α-[^2^H_6_]dihydroxycholestan-6-one ([^2^H_6_]-3β,5α-diHC-6O), cholestane-3β,5α,6β-triol (C-triol) and [^2^H_6_]cholestane-3β,5α,6β-triol ([^2^H_6_]-C-triol) were synthesised as previously described [16]. 4α- and 4β-hydroxyholesterols (4α-HC, 4β-HC), synthesised as described in [17], were kindly donated by G. Lizard, Université de Bourgogne. All other deuterated and non-deuterated oxysterols were from Avanti Polar Lipids (Alabaster, AL).

### 2.3 LC–MS analysis

Oxysterols were analysed as their Girard P (GP) derivatives using deuterium labelled internal standards as described in Griffiths et al. and Crick et al. [18,19]. In brief, 100 μL of plasma was added to 1.05 mL of ethanol containing deuterated internal standards 24S-[^2^H_7_]hydroxycholesterol, 22R-[^2^H_7_]hydroxycholesterol, 22R-[^2^H_7_]hydroxycholest-4-en-3-one, 7α-[^2^H_7_]hydroxycholesterol, 7α,25-[^2^H_6_]dihydroxycholesterol and [^2^H_7_]cholesterol (Avanti). The solution was diluted to 70% ethanol and centrifuged. The supernatant (1.5 mL 70% ethanol) was loaded on a Sep-Pak tC_18_ 200 mg cartridge (Waters, Elstree, UK) and the flow-through and a 5.5 mL wash with 70% ethanol combined. This fraction, the oxysterol fraction, was dried under reduced pressure, re-constituted in 100 μL of propan-2-ol and treated with KH_2_PO_4_ buffer (1 mL 50 mM, pH 7) containing 3 μL of cholesterol oxidase (2 mg/mL in H_2_O, 44 units/mg protein) for 1 h at 37 °C. Methanol (2 mL), glacial acetic acid (150 μL) and GP reagent (150 mg, 0.8 mmole) were added and the mixture incubated at room temperature over night. To remove excess derivatisation agent the reaction mixture was applied to a 60 mg OASIS HLB cartridge (Waters). A re-cycling protocol was adopted where the eluate is diluted with an equal volume of water and re-cycled on the column until the eluate is 17.5% methanol (19 mL). After a wash with 10% methanol (6 mL) GP-derivatised oxysterols were eluted in methanol (2 mL).

In contrast to sample preparation for GC–MS analysis, hydrolysis was not performed for LC–MS, hence non-esterified oxysterols were measured by LC–MS while total oxysterols by GC–MS.

### 2.4 Statistics

Paired sample *t* tests were performed. *P* < 0.05 was considered statistically significant. ^∗^*P* < 0.05; ^∗∗^*P* < 0.01.

## 3 Results

### 3.1 GC–MS

**Fig. 1:**
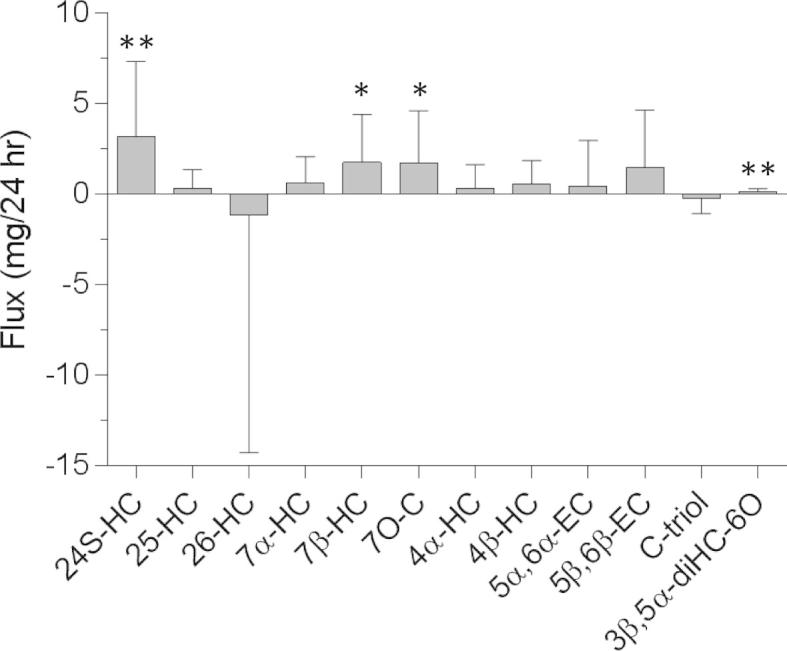
Flux of sterols out from (positive value) and into (negative value) brain. Plasma concentrations of sterols in the jugular vein and a forearm vein taken from coronary heart disease patients (*n* = 18) undergoing on-pump myocardial revascularization surgery were measured by GC–MS. The flux of sterol out from and into brain was calculated based on jugular vein and forearm vein differences assuming a flow of plasma to the brain of 450 mL/min. Paired sample *t* tests were performed. ^∗^*P* < 0.05; ^∗∗^*P* < 0.01.

Using GC–MS we measured the levels of 12 oxysterols in the jugular vein and in a vein in the forearm. Of these, we found statistical differences in the levels of 7β-hydroxycholesterol (7β-HC, *P* < 0.05), 7-oxocholesterol (7-OC, *P* < 0.05), 3β,5α-diHC-6O (*P* < 0.01) and 24S-HC (*P* < 0.01) corresponding to a flux of about 2, 2, 0.1 and 3 mg/24 h out from brain, assuming a flow of plasma of 450 mL/min through brain [6] (Fig. 1 and Supplementary data Table S1).

### 3.2 LC–MS

**Fig. 2:**
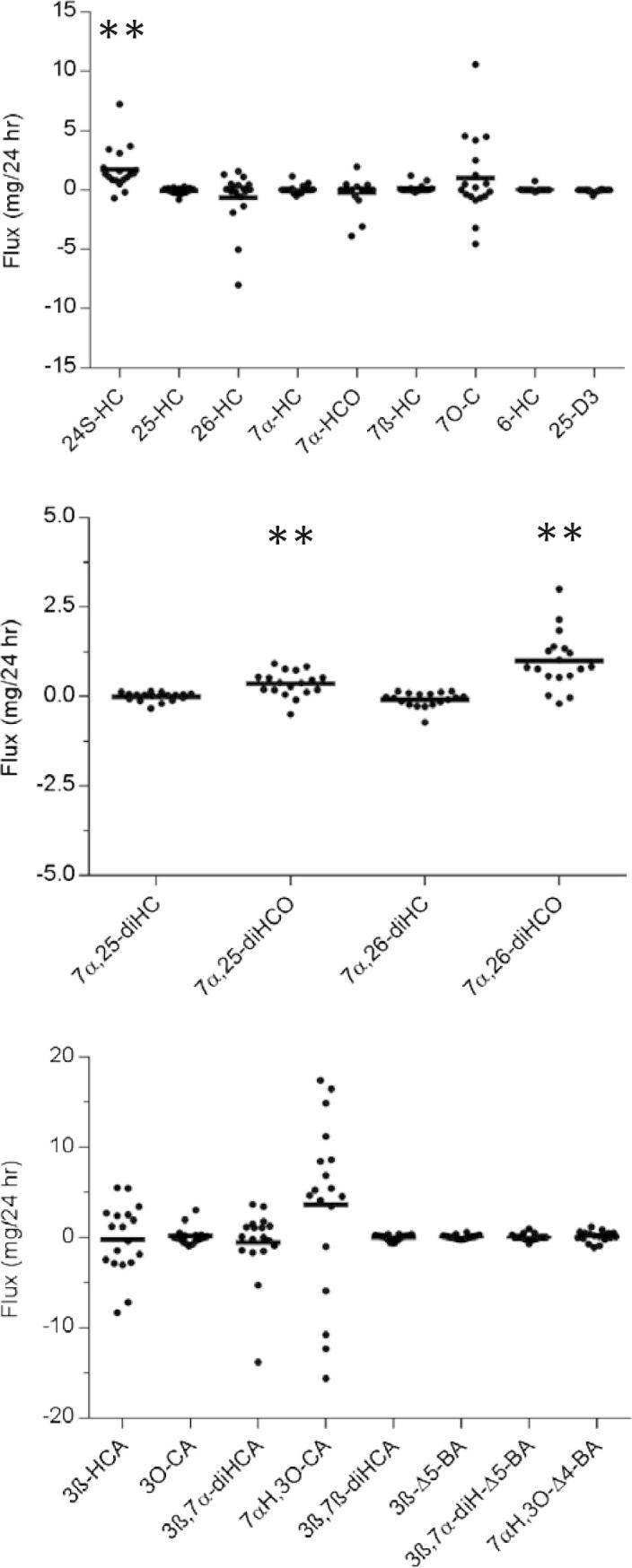
Flux of sterols out from (positive value) and into (negative value) brain. Plasma concentrations of sterols in the jugular vein and a forearm vein taken from coronary heart disease patients (*n* = 18) undergoing on-pump myocardial revascularization surgery were measured by LC–MS. The flux of sterol out from and into brain was calculated based on jugular vein and forearm vein differences assuming a flow of plasma to the brain of 450 mL/min. Individual data points are represented by filled circles. Mean values are indicated by a solid bar. Paired sample *t* tests were performed. ^∗^*P* < 0.05; ^∗∗^*P* < 0.01. Data for 7α,25-diHCO and 7α,26-diHCO has been reported previously [19]. See Supporting information Table S2 for a full list of all compound abbreviations.

Using LC–MS we similarly measured the levels of 20 cholesterol metabolites and also 25-hydroxyvitamin D_3_ (25-D_3_). An important methodological difference between the LC–MS and the GC–MS analysis was that only non-esterified metabolites were measured by LC–MS. We found a statistical difference in the concentration of 24S-HC (*P* < 0.01) between the two veins, corresponding to a flux from brain of about 2 mg/24 h. In an earlier report we noted that 7α,25-dihydroxycholest-4-en-3-one (7α,25-diHCO, *P* < 0.01) and 7α,(25R)26-dihydroxycholest-4-en-3-one (7α,26-diHCO, *P* < 0.01) were similarly exported from brain at a rate of about 0.5 and 1 mg/24 h, respectively [19] (Fig. 2 and Supplementary data Table S2).

## 4 Discussion

As mentioned in Section 1, there have been three previous studies investigating the flux of oxysterols from brain. Lütjohann et al. measured differences in oxysterol levels in plasma from the jugular vein and a brachial artery from 8 volunteers and found the flux of 24-HC out from brain into the circulation to be about 4 mg/24 h [3]. Björkhem et al. and Heverin et al. similarly measured the flux of oxysterols from brain from an additional 12 healthy subjects and also found 24-HC to be exported at a rate of about 6 mg/24 h, while 26-HC was imported to brain at a rate of about 4–5 mg/24 h [4,6]. In a further study, Meaney et al. measured the levels of cholestenoic acids in the jugular vein and brachial artery from 9 healthy subjects and found that 7αH,3O-CA is exported at a rate of about 2 mg/24 h from brain.

In the current study we have measured the differences in concentration of 18 oxysterols, 5 cholestenoic and 3 cholenoic acids between the jugular vein and a forearm vein taken from 18 coronary heart disease patients undergoing on-pump myocardial revascularization surgery. We find that 24S-HC is exported from brain at a rate of 2–3 mg/24 h.

Three other oxysterols, 7β-HC, 7O-C and 3β,5α-diHC-6O, were exported from brain at rates of about 2–0.1 mg/24 h. These differences were only observed when total oxysterols were measured. These three compounds are considered to be formed from cholesterol through reactive oxygen species (ROS) and might indicate oxidative stress activity in the brain. In particular, 3β,5α-diHC-6O can be formed chemically by ozone or via ozone-like mechanisms [20–22] in a pathway that might occur in the brain [23]. However, recent evidence supports the occurrence of an enzymatic-mediated mechanism of 3β,5α-diHC-6O formation using C-triol as a substrate (unpublished data). Since C-triol is formed by epoxide hydrolase that uses 5,6-epoxides as substrates [24], 3β,5α-diHC-6O may provide a specific form of transport out of the brain for oxidative stress-derived cholesterol epoxides.

Although not showing statistical significance both GC–MS analysis for total oxysterols and LC–MS analysis for non-esterified metabolites indicate import of 26-HC to brain. LC–MS analysis for 7αH,3O-CA indicate that this metabolite is exported from brain (Fig. 2). This result was observed for 13 of the 18 patients studied.

**Fig. 3:**
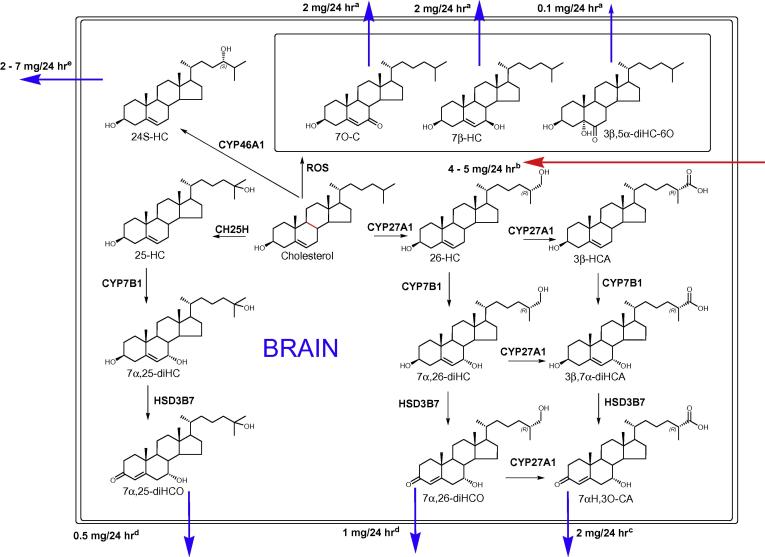
Cholesterol metabolism in brain. Cholesterol metabolites leaving and entering brain are indicated by arrows. Enzymes involved in cholesterol metabolism are shown. The metabolites within the boxed inset are likely formed through ROS. ^a^Present work. Formed via ROS. ^b^From reference [6]. ^c^From Ref. [9]. ^d^From Ref. [19]. ^e^From Refs. [3–6] and present work.

We recently reported that two other cholesterol metabolites 7α,25-diHCO (16/18 patients) and 7α,26-diHCO (16/18 patients) were exported from brain at rates of about 0.5 and 1 mg/24 h (Fig. 2) [19]. Therefore our data and that of others indicate that there are in fact multiple routes for export of cholesterol metabolites from brain (Fig. 3). The major exported oxysterol is 24S-HC, this is formed from cholesterol in a CYP46A1 catalysed reaction. CYP46A1 is known to be expressed in brain [25]. Other exported oxysterols include 7β-HC, 7O-C and 3β,5α-diHC-6O. These oxysterols are likely to be formed from cholesterol by ROS [21]. On the other hand, 26-HC is imported from the circulation into brain [6]. In brain 26-HC can be metabolised via the “acidic pathway” of bile acid biosynthesis [26]. One branch of this pathway is via 7α,(25R)26-dihydroxycholesterol (7α,26-diHC) and 7α,26-diHCO leading to 7αH,3O-CA. The latter two compounds are found to be exported from brain [9,19]. 7α,26-diHC is formed from 26-HC via the enzyme CYP7B1, known to be expressed in brain [27], and then converted to 7α,26-diHCO by the enzyme HSD3B7 and ultimately to 7αH,3O-CA via CYP27A1 [26]. CYP7B1 and HSD3B7 similarly metabolise 25-HC through to 7α,25-dihydroxycholesterol (7α,25-diHC) and ultimately to the export product 7α,25-diHCO, respectively. The second branch of the acidic pathway from 26-HC involves further oxidation by CYP27A1 to generate 3β-hydroxycholest-5-en-(25R)26-oic acid (3β-HCA) followed by 7α-hydroxylation via CYP7B1 to give 3β,7α-dihydroxycholest-5-enoic acid (3β,7α-diHCA). Neither of these latter metabolites are exported from brain.

A limitation of the current study is that the patients took several mediations that, potentially, could have affected the results. Having found, however, both enzymatic and non-enzymatic oxysterols are exported from the brain, a potential drug-derived bias seems unlikely, although one cannot be ruled out. In addition, results on 24S-HC are confirmatory of previous studies carried out in healthy volunteers [3,4,6]. On the other hand, the data of the present study were obtained in patients with coronary heart disease (CHD) and might not apply to other clinical settings or to healthy individuals. Notably, the efflux of oxysterols pertinent to the oxidative stress pathway may be associated with brain injury dependent on alterations of the cerebral vasculature likely to be present in patients with CHD. If this hypothesis holds true these oxysterols would provide a link in support of the theory of vascular-driven neurodegeneration [28].

## Appendix A Supplementary data

Supplementary data 1 This document contains supplementary tables.
