# Supplementary material for: Cholesterol metabolites exported from human brain
Source: Steroids. 2015 Jul;99:189–93. doi: 10.1016/j.steroids.2015.01.026 (PMC4503873; doi:10.1016/j.steroids.2015.01.026)
Supplement: Supplementary data 1 — This document contains supplementary tables. [file mmc1.docx]

Table S1. Plasma concentrations of sterols determined by GC-MS in the jugular vein and a forearm vein taken from coronary heart disease patients (n = 18) undergoing on-pump myocardial revascularization surgery. The flux of sterol out of the brain (positive value) and into brain (negative value) is calculated based on a flow of plasma to the brain of 450 mL/min. Mean concentrations ± standard deviation (ng/mL ± SD) and flux (mg/24 hr) values are given. Total sterols comprising the sum of esterified and non-esterified sterols were measured.

| Systematic name  (common name, abbreviation) | Jugular vein  ng/mL ± SD | Peripheral vein  ng/mL ± SD | Difference  ng/mL | Flux  mg/24hr | t test  P value |
| --- | --- | --- | --- | --- | --- |
| Cholest-5-ene-3β,24S-diol  (24S-hydroxycholesterol, 24S-HC) | 38.09±18.30 | 33.18±13.81 | 4.90 | 3.18 | 0.005  ** |
| Cholest-5-ene-3β,25-diol  (25-hydroxycholesterol, 25-HC) | 6.31±2.21 | 5.83±2.32 | 0.48 | 0.31 | 0.227  NS |
| Cholest-5-ene-3β,(25R)26-diol  ((25R)26-Hydroxycholesterol, 26-HC)^a^ | 41.93±28.05 | 43.67±30.61 | -1.74 | -1.13 | 0.72  NS |
| Cholest-5-ene-3β,7α-diol  (7α-Hydroxycholesterol, 7α-HC) | 9.97±11.39 | 8.99±9.34 | 0.97 | 0.63 | 0.081  NS |
| Cholest-5-ene-3β,7β-diol  (7β-Hydroxycholesterol, 7β-HC) | 15.75±15.03 | 13.05±11.26 | 2.71 | 1.75 | 0.012  * |
| 3β-Hydroxycholest-5-en-7-one  (7-Oxocholesterol, 7O-C) | 13.41±9.32 | 10.74±6.12 | 2.67 | 1.73 | 0.021  * |
| Cholest-5-ene-3β,4α-diol  (4α-Hydroxycholesterol, 4α-HC) | 5.40±2.58 | 4.86±1.82 | 0.54 | 0.35 | 0.269  NS |
| Cholest-5-ene-3β,4β-diol  (4β-Hydroxycholesterol, 4β-HC) | 13.21±6.90 | 12.37±7.32 | 0.85 | 0.55 | 0.093  NS |
| 3β-Hydroxycholestan-5α,6α-epoxide  (5α,6α-Epoxycholesterol, 5α,6α-EC) | 9.25± 4.11 | 8.57± 4.36 | 0.68 | 0.44 | 0.47  NS |
| 3β-Hydroxycholestan-5β,6β-epoxide  (5β,6β-Epoxycholesterol, 5β,6β-EC) | 20.99±17.18 | 18.74±14.76 | 2.25 | 1.46 | 0.069  NS |
| Cholestan-3β,5α,6β-triol  (Cholestanetriol, C-triol) | 3.91± 1.90 | 4.24± 1.46 | -0.33 | -0.22 | 0.296  NS |
| 3β,5α-Dihydroxycholestan-6-one  (5α-Hydroxy-6-oxocholesterol, 3β,5α-diHC-6O) | 1.31± 0.48 | 1.11± 0.48 | 0.19 | 0.13 | 0.007  ** |

Paired sample *t* tests were performed. * P < 0.05; ** P < 0.01. NS not significant.

^a^ Also know by the non-systematic name 27-hydroxycholesterol.

Table S2. Plasma concentrations of sterols determined by LC-MS in the jugular vein and a forearm vein taken from coronary heart disease patients (n = 18) undergoing on-pump myocardial revascularization surgery. The flux of sterol out of the brain (positive value) and into brain (negative value) is calculated based on a flow of plasma to the brain of 450 mL/min. Mean concentrations ± standard deviation (ng/mL ± SD) and flux (mg/24 hr) values are given. Non-esterified sterols only were analysed.

| Systematic name  (common name, abbreviation) | Jugular vein  ng/mL ± SD | Peripheral vein  ng/mL ± SD | Difference  ng/mL | Flux  mg/24hr | t test  P value |
| --- | --- | --- | --- | --- | --- |
| Cholest-5-ene-3β,24S-diol  (24S-hydroxycholesterol, 24S-HC) | 9.67±4.94 | 7.00±3.15 | 2.67 | 1.73 | 0.001  ** |
| Cholest-5-ene-3β,25-diol  (25-hydroxycholesterol, 25-HC) | 1.15±0.65 | 1.25±0.82 | -0.09 | -0.06 | 0.317  NS |
| Cholest-5-ene-3β,(25R)26-diol  ((25R)26-Hydroxycholesterol, 26-HC) | 10.65±4.91 | 11.63±7.37 | -0.98 | -0.63 | 0.270  NS |
| Cholest-5-ene-3β,7α-diol  (7α-Hydroxycholesterol, 7α-HC) | 0.39±0.57 | 0.33±0.41 | 0.06 | 0.04 | 0.650  NS |
| 7α-Hydroxycholest-4-en-3-one  (7α-HCO) | 0.43±0.85 | 0.88±2.09 | -0.45 | -0.29 | 0.359  NS |
| Cholest-5-ene-3β,7β-diol  (7β-Hydroxycholesterol, 7β-HC) | 0.53±0.81 | 0.33±0.61 | 0.20 | 0.13 | 0.125  NS |
| 3β-Hydroxycholest-5-en-7-one  (7-Oxocholesterol, 7O-C) | 5.13±5.36 | 3.58±4.00 | 1.54 | 1.00 | 0.225  NS |
| Cholest-4-ene-3β,6-diol or Cholest-5-ene-3β,6-diol (6-Hydroxycholesterol, 6-HC)^a^ | 0.33±0.62 | 0.28±0.48 | 0.05 | 0.03 | 0.488  NS |
| 9,10-Secocholesta-5Z,7E,10-trien-3β,25-diol (25-hydroxyvitamin D_3_, 25-D_3_)^b^ | 0.59±0.61 | 0.74±0.76 | -0.15 | -0.10 | 0.113  NS |
| Cholest-5-ene-3β,7α,25-triol (7α,25-Dihydroxycholesterol, 7α,25-diHC) | 0.35±0.19 | 0.35±0.13 | 0.00 | 0.00 | 0.999  NS |
| 7α,25-Dihydroxycholest-4-en-3-one (7α,25-diHCO)^c^ | 2.02±1.37 | 1.46±1.29 | 0.56 | 0.36 | 0.000  ** |
| Cholest-5-ene-3β,7α,26-triol (7α,26-Dihydroxycholesterol, 7α,26-diHC) | 0.47±0.32 | 0.60±0.34 | -0.13 | -0.09 | 0.103  NS |
| 7α,26-Dihydroxycholest-4-en-3-one (7α,26-diHCO)^c^ | 3.76±1.24 | 2.22±1.04 | 1.54 | 1.00 | 0.000  ** |
| 3β-Hydroxycholest-5-en-(25R)26-oic acid (3β-HCA) | 49.90±23.88 | 50.26±25.17 | -0.36 | -0.23 | 0.803  NS |
| 3-Oxocholest-4-en-(25R)26-oic acid (3O-CA) | 3.87±3.05 | 3.65±3.13 | 0.22 | 0.14 | 0.538  NS |
| 3β,7α-Dihydroxycholest-5-en-26-oic acid (3β,7α-diHCA) | 17.70±10.69 | 18.57±10.29 | -0.87 | -0.56 | 0.549  NS |
| 7α-Hydroxy-3-oxocholest-4-en-26-oic acid (7αH,3O-CA) | 56.31±21.62 | 50.68±19.90 | 5.63 | 3.65 | 0.124  NS |
| 3β,7β-Dihydroxycholest-5-en-26-oic acid (3β,7β-diHCA) | 2.73±1.32 | 2.72±1.27 | 0.01 | 0.01 | 0.943  NS |
| 3β-Hydroxychol-5-en-24-oic acid (3β-Δ5-BA) | 1.13±1.11 | 1.03±0.99 | 0.10 | 0.06 | 0.212  NS |
| 3β,7α-Dihydroxychol-5-en-24-oic acid (3β,7α-diH-Δ5-BA) | 1.04±2.27 | 1.00±2.11 | 0.04 | 0.03 | 0.751  NS |
| 7α-Hydroxy-3-oxochol-4-en-24-oic acid (7αH,3O-Δ4-BA) | 1.97±1.01 | 1.77±1.24 | 0.20 | 0.13 | 0.376  NS |

Paired sample *t* tests were performed. * P < 0.05; ** P < 0.01. NS not significant.

^a^ Cholest-4-ene-3β,6-diol and cholest-5-ene-3β,6-diol can be formed from 3β-hydroxycholestan-5,6-epoxides via hydration and subsequent dehydration during sample preparation.

^b^ Values for 25-D_3_ are quantitative estimates.

^c^ From reference [19].
